# Effect of Participation with Accompanying Household Member in the Complete Health Improvement Program in Appalachia

**DOI:** 10.1155/2019/9648926

**Published:** 2019-01-29

**Authors:** Dhatri Kotekal, Melanie Worley, Hemal Patel, Laura Jensen, Godwin Y. Dogbey, David Drozek

**Affiliations:** ^1^Ohio University Heritage College of Osteopathic Medicine, Athens, OH 45701, USA; ^2^Summa Akron City Hospital, Akron, Ohio 44304, USA; ^3^School of Osteopathic Medicine, Campbell University, NC 27506, USA; ^4^Department of Specialty Medicine, Ohio University Heritage College of Osteopathic Medicine, Athens, OH 45701, USA

## Abstract

Intensive therapeutic lifestyle modification programs, such as the Complete Health Improvement Program (CHIP), reduce cardiovascular disease (CVD) risk factors. However, there are little data on how participation in CHIP with a household member can affect CVD biomarkers. This study focuses on the benefit of joint participation of household members in CHIP in order to have a better outcome in improving CVD risk factors compared with lone or individual participation. Data from 20 CHIP classes offered from 2011 to 2015 in Athens, Ohio, where each class was conducted over 2-4 months, consisting of 16-18 sessions, were collected. Body mass index (BMI), blood pressure, fasting glucose, and lipid profiles were measured before and near the completion of each class. A statistically significant greater reduction in BMI (*p* = 0.003) in those who attended with a household member compared to those who attended as individuals was found. CHIP has some effect on various CVD risk factors for those who attend intensive therapeutic lifestyle modification programs with an accompanying household member. Hence, encouragement of participation with a family member or a “buddy” may be prudent, especially if weight reduction is a key program participation goal. Further evaluation of the “buddy effect” involving both of those residing in the same household and those who do not but nevertheless provide mutual support is warranted.

## 1. Introduction

Cardiovascular disease (CVD) is the leading cause of death in the United States [1]. Risk factors for CVD include dyslipidemia, hypertension, smoking, elevated body mass index (BMI), and diabetes [2–4]. Intensive therapeutic lifestyle modification programs (ITLMP) have been effective in improving CVD biomarkers and risk factors [5–7]. One well-studied ITLMP is the Complete Health Improvement Program (CHIP), which has demonstrated short-term effectiveness in improving many CVD risk factors [8, 9].

The Appalachian region of the United States has consistently been associated with high morbidity and mortality resulting from chronic diseases such as CVD and diabetes, lack of access to health care, and higher rates of uninsured people [10]. Prior studies have demonstrated that CHIP was effective in reducing CVD risk factors in an Appalachian population [11, 12]. In spite of the growing literature on ITLMP and CVD risk factors, there is a paucity of studies evaluating the effect of participation with an accompanying household member.

Household members may express similarities in lipid profiles due to genetic factors and similar living environments [13]. Furthermore, the resemblance in the physical health profile characteristics in systolic blood pressure and BMI of a family predisposes the development of similar CVD risk factor patterns [14]. Family can influence positive changes, such as increased physical activity seen in spouses participating together in lifestyle change programs [15]. A study evaluating environmental versus genetic influence of acquiring CVD in family members has shown that environment plays a significant role in developing CVD risk factors [16].

This study aimed to see if better CVD risk factor outcomes were achieved by participating in an ITLMP with a household member as compared to attending alone. It was hypothesized that participation with an accompanying household member would be associated with better outcomes than participation alone.

## 2. Methods

### 2.1. Study Participants

Participants attended CHIP classes offered from 2011 to 2015 in Athens, Ohio, a rural Appalachian college town. For recruitment, the program was promoted via the local media, health care providers, and churches. Participants came from varied socioeconomic backgrounds. As part of the registration process for CHIP, participants were asked to sign a consent statement to allow the use of their deidentified aggregated data for research purposes. They were informed that their choice to opt out of participation would not alter their eligibility to participate in CHIP. Data were stored on password-protected devices with restricted access to only approved CHIP administrators and study investigators. Approval for the study was obtained from Live Healthy Appalachia, the local CHIP administrator, and the Ohio University Institutional Review Board.

### 2.2. CHIP Description

The CHIP intervention consisted of 16 to 18 two-hour group sessions that were provided over 4 to 8 weeks. A typical session consisted of an instructional video, group discussion, cooking demonstrations, and an exercise component [9]. The goal of CHIP was for participants to consume plant-based whole foods, such as minimally processed vegetables, fruits, whole grains, legumes, and nuts. This was done through fostering self-care and awareness of lifestyle habits. Specifically, overall dietary fat was to be kept below 20% of total calories, daily intake of sugar less than 10 teaspoons, salt intake less than 2000 mg, cholesterol below 50 mg, and fiber intake 35 to 40 grams. Stress management techniques were taught and encouraged for daily use. Daily exercise of at least 30 minutes of moderate activity or 10,000 steps measured using a pedometer was encouraged. Strength training and resistance exercises were encouraged for 20-30 minutes, 2-3 days per week.

### 2.3. Data Collection and Reporting

Demographic data were collected as well as status of participation with a household member (sharing the same physical address) or alone, as an individual participant, without a household member. Biomedical assessments were made at baseline and before session 12, near the end of the program. Assessments included a lifestyle questionnaire to evaluate dietary and exercise habits as well as current illnesses and medications, weight, height, pulse rate, and blood pressure. BMI was calculated as weight in kilograms divided by the square of height in meters with a normal range considered as 18.5-24.9, overweight 25.0-29.9, and obese category 30 or above [17]. Blood was collected via venipuncture by trained phlebotomists to determine total cholesterol (TC), low density lipoprotein cholesterol (LDL), high density lipoprotein cholesterol (HDL), triglyceride (TG), and fasting blood glucose (GLU) levels. The data were entered into a password-protected Microsoft Access based database at the Live Healthy Appalachia office.

### 2.4. Statistical Analysis

Actual and percentage changes in the biomarker outcome variables during the CHIP program were computed. Participation in the program as an individual or alone (without a household member) or with an accompanying household member was the variable of interest. These were considered two independent groups defined as “Individual” for those who participated in the lifestyle program solely on their own and “Household” for those who participated with an accompanying member of their household. Data from both participating household members were used in the study analysis such that each household member was considered an independent participant in the “Household” group. Independent samples* t*-tests were conducted to compare differences between those attending with a household member (Household) and those attending alone (Individual) with respect to the mean changes in their biomarker outcomes. All differences were considered statistically significant if* p* ≤ 0.05.

## 3. Results

The demographic characteristics of the CHIP participants in this study are shown in Table 1. In all, 512 people participated in the program over the period under study. Of this, 66.4% participated as an individual, or alone, while 33.6% participated with an accompanying household member. The average age of all the participants was 53.19 (±12.6 standard deviation (SD)) years with a range of 19 through 82 years.

Baseline averages were calculated before CHIP intervention and after for both Individual and Household groups. All biomarkers demonstrated decreased values in both groups, after completion of the program, as shown in Table 2.

As shown in Table 3, the only cardiovascular risk factor change that was statistically significant between the Individual and the Household groups was BMI. The reduction in BMI was higher for Household than the Individual participant group.

## 4. Discussion

One of the remarkable results from this study was an improvement in all CVD risk factors from baseline, for both those who participated in CHIP alone and those with an accompanied household member. This observation was consistent with results from multiple studies involving populations similar to that in this study and other populations [8, 9, 11]. For example, the English Longitudinal Study of Aging (ELSA) reported that positive behavioral changes in one spouse strongly influenced the other partner to make changes resulting in correlations with increased physical activity (*r* = 0.478) and reduced weight (*r* = 0.311) [18]. Furthermore, Mosca et al. demonstrated that, with the support of family members, patients who were hospitalized with CVD benefited from lifestyle intervention at a 1-year follow-up, with an improvement in diet score (*p* = 0.04) and likelihood to exercise at least 3 days per week (*p* = 0.04) [19]. Lastly, a study utilizing Partners Together in Health intervention model for encouraging healthy eating behaviors after cardiac rehabilitation reported increased long-term adherence as compared to the usual individual care [16].

This current study revealed that those who participated in CHIP with a household member (i.e., spouse, sibling, parent, or child) showed improvement in BMI. This would suggest that familial support was important in the attainment of at least one healthy lifestyle goal. Such familial support could arise from a shared environment where food preparation and exercise are done together. It could also be through extrinsic motivation whereby the participant is held individually and jointly accountable to achieve the necessary health goals. It may be especially effective if the individuals who purchase and prepare food for the household participate in lifestyle modification as well.

To contextualize by quantifying the BMI improvement observed in this study, it should be noted that BMI improved in 4-8 weeks by a 1.29% mean change with household participation as compared to 1.09% for participation alone, as a solo individual (p=0.003). With the average BMI in men and women in the U.S. being 26.6 and 26.5, respectively, an improvement of 1.29% could mean a loss of 5-10 pounds [17]. Greater BMI is associated with higher total cholesterol, lower HDL, higher blood pressure, and diabetes, all critical biomarkers for cardiovascular disease [20]. Reduction in BMI was significantly larger when participating in CHIP with a household member, which may in turn play a role in indirectly reducing the other biomarkers and make for a more effective lifestyle modification program.

However, in this study, outcomes such as TC, HDL, LDL, TG, BPSYS and BPDIA, did not significantly change from their baseline values. Perhaps a longer length of intervention of follow up, or greater mean change in BMI is needed before significant changes are seen in other biomarkers. For example, in the Look AHEAD trial, larger weight losses produced greater improvements in HbA1c, systolic blood pressure, HDL, and triglycerides at years 1 and 4 of follow up [21].

### 4.1. Limitations

A confounding variable to consider is how different genders respond to lifestyle change. The participants in this study were overwhelmingly women, comprising 73.8% of the total sample, and could possibly skew the results, as men are known to improve CVD risk factors better with lifestyle change [22, 23]. The first reported CHIP intervention study, a hospital-based program conducted in Kalamazoo, Michigan (*n* = 288), demonstrated that males with the highest levels of TC at program entry (i.e., 240-279 mg/dL) experienced a 22% reduction in 30 days, whereas females with the highest levels of TC experienced a mean decrease of 11% [22]. These findings are consistent with a study conducted in CHIP Australasian study in which men showed improved responsiveness to reductions in chronic disease risk factors [23].

Furthermore, it may have been that those who participated with a household member were more enthusiastic, leading to recruitment of the household member. The enthusiasm, rather than the presence of the household member, may have been a major factor in success.

Canning et al. demonstrated a relationship between age and cardiovascular disease risk factors (*p*=.049). They showed that increased BMI was associated with an increase in prevalent risk in younger (18-40 year olds) and middle age (40-65 years old) groups but not the older age (65+ years old) groups [24]. These differences in responsiveness to lifestyle change based on gender and age could further explain why the other CVD risk factors were not significantly changed from baseline. The findings of this current study may serve to inform the medical community regarding potential outcomes associated with lifestyle modification and cardiovascular health.

The results of this study may actually underrepresent the effect of household member support. This may arise from family members participating that do not share the same physical address and were not captured as household members. In addition, others may participate with close friends who provide support. A future study could be designed to better evaluate the “buddy effect” by including these other groups in the analysis.

## 5. Conclusion

CHIP has been shown to have effects on various cardiovascular disease risk factors among residents of rural Appalachia. Those participating with a household member elicited a higher reduction in BMI compared to those who participated alone, as solo individuals. Encouragement of participation with a family member or a “buddy” may be prudent, especially if weight reduction is a goal of the program or participants. Further evaluation of the “buddy effect” involving both those residing in the same household and those who do not but nevertheless provide mutual support, is warranted.

## Figures and Tables

**Table 1:** Demographic characteristics of the CHIP study participants.

**Characteristics**	

Participation Group, *n* (%)	**512**
Individual	340 (66.4)
Household	172 (33.6)
Gender, *n* (%)	**512**
Male	134 (26.2)
Female	378 (73.8)

**(a):** 

**Risk Factor**	***n***	**Baseline Average for Individual Group BEFORE**	**Average for Individual Group AFTER**

TC mg/dL	340	189.2	170.7
HDL mg/dL	340	50.2	46.0
LDL mg/dL	336	114.6	100.3
TG mg/dL	341	127.1	123.2
GLU mg/dL	337	105.5	99.2
BMI kg/m^2^	321	32.3	31.3
BPSYS mmHg	320	129.6	123.8
BPDIA mmHg	322	77.8	76.1
PULSE beats per minute	296	75.7	71.0

**(b):** 

**Risk Factor**	***n***	**Baseline Average for Household Group BEFORE**	**Average for Household Group AFTER**

TC mg/dL	172	182.9	161.0
HDL mg/dL	172	46.2	42.2
LDL mg/dL	169	111.0	94.9
TG mg/dL	172	131.1	121.3
GLU mg/dL	172	104.3	99.4
BMI kg/m^2^	168	31.6	30.2
BPSYS mmHg	168	129.3	122.0
BPDIA mmHg	168	77.8	75.0
PULSE beats per minute	158	72.9	70.4

**Table 3:** Biomarker outcomes for CHIP participation alone (Individual) or with an accompanying household member (Household). *Abbreviations.* Standard deviation (SD), total cholesterol (TC), high density lipoprotein (HDL), triglycerides (TG), fasting blood glucose (GLU), body mass index (BMI), systolic blood pressure (BPSYS), and diastolic blood pressure (BPDIA).

**Risk Factors**	**Individual Group**			**Household Group**			***P*** **value**
	***n***	**Mean Change, (**%**)**	**SD**	***n***	**Mean Change, (**%**)**	**SD**	

TC	340	-18.6 (9.8)	25.4	172	-21.9 (12.0)	27.4	.177
HDL	340	-4.3 (8.5)	6.8	172	-4.02 (8.7)	7.4	.717
LDL	339	-14.5 (11.4)	21.5	169	-16.56 (14.8)	23.2	.320
TG	340	-3.9 (3.7)	48.1	172	-9.84 (7.5)	51.5	.198
GLU	337	-6.32 (5.9)	24.1	172	-4.9 (4.7)	11.1	.468
BMI^∗^	328	-1.02 (3.2)	1.1	168	-1.29 (4.1)	0.8	.003
BPSYS	322	-5.71 (4.4)	14.6	168	-7.34 (5.7)	14.1	.238
BPDIA	322	-1.76 (2.3)	9.7	168	-2.8 (3.6)	10.1	.267
PULSE	296	-4.47 (5.9)	13.2	158	-2.5 (3.5)	10.1	.107

*Note.* ^∗^Statistically significant at .01 level.

## Data Availability

The raw data used to support the findings of this study are available from the corresponding author upon request.
